# Assessing the role of the private sector in surveillance for malaria elimination in Haiti and the Dominican Republic: a qualitative study

**DOI:** 10.1186/s12936-019-3024-3

**Published:** 2019-12-05

**Authors:** Abigail Sidibe, Alysse Maglior, Carmen Cueto, Ingrid Chen, Arnaud Le Menach, Michelle A. Chang, Thomas P. Eisele, Katherine Andrinopolous, Joseph Cherubin, Jean Frantz Lemoine, Adam Bennett

**Affiliations:** 1 Malaria Elimination Initiative, The Global Health Group, University of California, San Francisco, CA USA; 2 Clinton Health Access Initiative, Washington, DC USA; 3 Malaria Branch, Division of Parasitic Diseases and Malaria, Center for Global Health, Centers for Disease Control and Prevention, Atlanta, GA USA; 4 School of Public Health and Tropical Medicine, Tulane University, New Orleans, LA USA; 5 MOSCTHA, Santo Domingo, Dominican Republic; 6 Ministry of Public Health and Population, Port-au-Prince, Haiti

**Keywords:** Malaria, Haiti, Dominican Republic, Private health sector, Private sector engagement, Care-seeking behavior, Traditional healers, Migrant population

## Abstract

**Background:**

Haiti and the Dominican Republic (DR) are targeting malaria elimination by 2022. The private health sector has been relatively unengaged in these efforts, even though most primary health care in Haiti is provided by non-state actors, and many people use traditional medicine. Data on private health sector participation in malaria elimination efforts are lacking, as are data on care-seeking behaviour of patients in the private health sector. This study sought to describe the role of private health sector providers, care-seeking behaviour of individuals at high risk of malaria, and possible means of engaging the private health sector in Hispaniola’s malaria elimination efforts.

**Methods:**

In-depth interviews with 26 key informants (e.g. government officials), 62 private providers, and 63 patients of private providers, as well as 12 focus group discussions (FGDs) with community members, were conducted within seven study sites in Haiti and the DR. FGDs focused on local definitions of the private health sector and identified private providers for interview recruitment, while interviews focused on private health sector participation in malaria elimination activities and treatment-seeking behaviour of febrile individuals.

**Results:**

Interviews revealed that self-medication is the most common first step in the trajectory of care for fevers in both Haiti and the DR. Traditional medicine is more commonly used in Haiti than in the DR, with many patients seeking care from traditional healers before, during, and/or after care in the formal health sector. Private providers were interested in participating in malaria elimination efforts but emphasized the need for ongoing support and training. Key informants agreed that the private health sector needs to be engaged, especially traditional healers in Haiti. The Haitian migrant population was reported to be one of the most at-risk groups by participants from both countries.

**Conclusion:**

Malaria elimination efforts across Hispaniola could be enhanced by engaging traditional healers in Haiti and other private providers with ongoing support and trainings; directing educational messaging to encourage proper treatment-seeking behaviour; and refining cross-border strategies for surveillance of the high-risk migrant population. Increasing distribution of rapid diagnostic tests (RDTs) and bi-therapy to select private health sector facilities, accompanied by adopting regulatory policies, could help increase numbers of reported and correctly treated malaria cases.

## Background

Haiti and the Dominican Republic (DR) are the only remaining countries in the Caribbean with endemic malaria transmission [1]. Although elimination of *Plasmodium falciparum* was almost achieved in the 1960s, malaria resurged in the 1970s following reductions in funding. In the past decade, a new wave of funding and ambition has reinvigorated global goals of malaria eradication. Recognizing that elimination on the island of Hispaniola will require a coordinated approach, the Haitian and Dominican National Malaria Control Programmes (NMCPs) developed a bi-national plan to eliminate malaria by the year 2020, which now has been extended to 2022 [2, 3]. However, malaria transmission and health care systems differ considerably between the two countries which posess unique challenges to elimination efforts. In 2017, the island of Hispaniola reported 19,476 cases, 98% (19,135 cases) of which were reported in Haiti and only 2% (341 cases) were reported in the DR [4]. Actual case burden is likely much higher, as many cases are not reported through the national surveillance systems.

In order to achieve malaria elimination on the island of Hispaniola, all malaria cases must be detected, documented, and treated. However, due to accessibility and preference, populations at high risk of malaria often seek malaria treatment in the private health sector which has remained largely unengaged in these efforts [2, 5]. Engaging private providers is critical for effective case management and reporting, especially in settings where the private health sector plays a large role [6].

The private health sector consists of formal institutions, such as pharmacies, clinics, and hospitals, as well as informal institutions, such as shops, itinerant drug vendors, and traditional healers. Informal private providers might not have received formal training and their clinical practice might not be registered or licensed by any governing body. Prior research has shown that private health sector involvement in malaria surveillance, diagnosis and treatment efforts has strengthened national malaria elimination programmes [7–12]. However, the participation of the private health sector in malaria surveillance and elimination efforts in Haiti and the DR is largely unexplored, as are the care-seeking behaviours of individuals at high risk of malaria (either for behavioural reasons—e.g. migrant agricultural workers; or more immunological reasons—e.g. pregnant women). This study describes the role of private providers in case management and surveillance, explores care-seeking behaviour of individuals at high risk of malaria, and investigates possible means of engaging the private health sector in Hispaniola’s malaria elimination efforts.

## Methods

### National health system context

#### Haiti

The provision of healthcare in Haiti is highly fragmented, which has resulted in limited services and poor quality [13]. The formal health sector is comprised of public, private non-profit (e.g. non-governmental organizations and religious organizations), “mixed” non-profit [private management with staff paid for by the Ministry of Public Health and Population (French acronym MSPP)] and private for-profit organizations [5, 14]. Of more than 900 health institutions, roughly 38% are public, 42% are private, and 20% are mixed [15]. Informal private health sector providers, such as traditional medicine practitioners, play a major role as well. An estimated 50–80% of the population use traditional medicine services, due to spiritual beliefs as well as lower cost and greater accessibility [5, 14, 16]. A 2017 census found that only 23% of Haitian households live within 5 kilometres of a primary care facility of good quality in the formal health sector (a “good quality” facility was defined as having a high overall score on four service delivery domains: accessible care, effective service delivery, management and organization, and primary care functions) [17].

Malaria cases are almost exclusively diagnosed by passive detection, as there is little active surveillance in the country. Rapid diagnostic tests (RDTs) were introduced in 2010, became national policy shortly thereafter, and are currently used to confirm cases in the national surveillance system at a ratio of 3:1 compared to microscopy [18]. In 2011, chloroquine-primaquine bi-therapy was adopted as first-line treatment [2]. Primaquine (PQ) is incorporated into bi-therapy for its gametocytocidal effect, although the absence of PQ during treatment with chloroquine does not render the treatment ineffective. Despite high utilization of the private health sector, only the public and mixed institutions have access to PQ to satisfy the bi-therapy strategy. However, even among these institutions the availability of PQ is low; only 31% of these formal institutions stocked PQ according to a 2013 government report [15].

#### Dominican Republic

Prior to 2001, the majority of the population in the Dominican Republic was theoretically covered by a public system financed by general taxes. This system encouraged growth of the formal private health sector financed by voluntary reimbursement insurance, prepaid payment plans, and out-of-pocket expenditures. In 2001, the private health sector’s importance increased following adoption of a new healthcare framework, consisting of a complex system of public, private, and non-profit institutions. As of 2012, there were 1853 public sector facilities (1703 primary care units and 150 s- and third-level treatment centres) [19], and an estimated 7121 private sector facilities (6818 for-profit facilities and 283 non-profit facilities) [20].

Malaria diagnosis is primarily done by microscopy. RDTs have been introduced in the past 5 years but are mainly used for active case detection and are not universally used in health facilities. First-line treatment is chloroquine-PQ bi-therapy, which is freely available in the public sector only [2]. As in Haiti, there is limited information on private health sector adherence to malaria case reporting and surveillance, despite the presence of a central-level database managed by the National Center for the Control of Tropical Diseases (Spanish acronym CENCET) where both passively and actively detected cases are supposed to be reported [19, 20].

### Selection of study sites and participants

#### Study sites

**Fig. 1:**
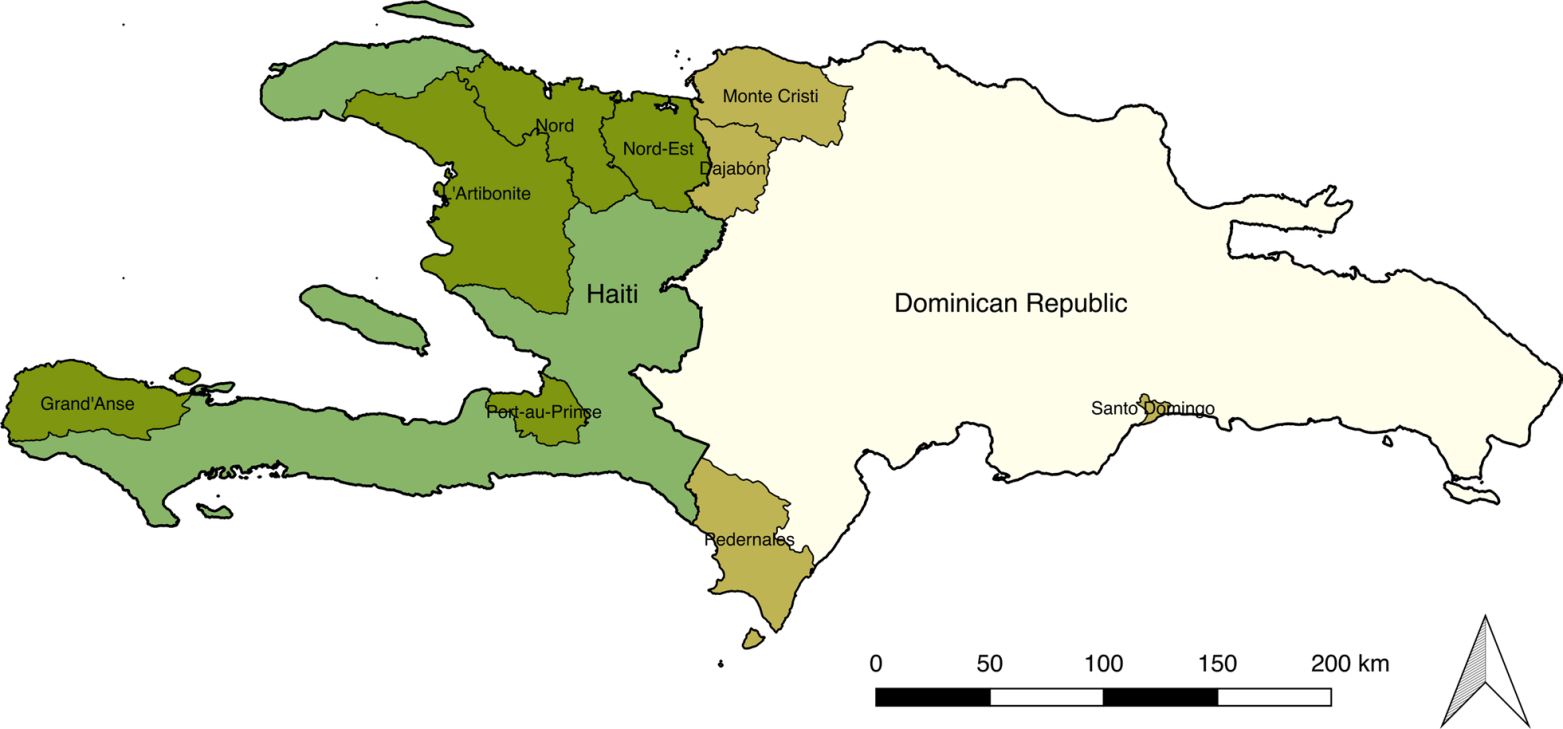
Study areas, Haiti and Dominican Republic, 2015

Study sites were selected due to their relatively high burden of malaria, reputation as high traffic areas for cross border migration, and/or presence of government and non-governmental organizations (Fig. 1). Study sites in Haiti were: Port-Au Prince (in the Ouest Department), Artibonite Department, Grande’Anse Department, and Nord/Nord-Est Departments. The Nord and Nord-Est Departments were combined into one effective study site at the recommendation of the local research assistants. Study sites in the DR were Dajabon Province, Monte Cristi Province, and Pedernales Province. A combination of purposive and snowball sampling methods were used to recruit community members for focus group discussions (FGDs), and key informants, private providers, and patients of private providers for in-depth interviews. All participants must have been 18 years of age or older.

#### Key informant interviews

Key informants were selected based on their involvement in malaria activities, such as NMCP staff, non-governmental organization staff, departmental government leaders. Key informants were recruited using purposive sampling through the following networks: Sociocultural Movement for Haitian Workers (Spanish acronym MOSCTHA), a private non-profit organization in the DR dedicated to advancing human rights of Haitian immigrants; staff at the International Training and Education Center for Health (I-TECH), a non-governmental organization in Haiti providing specialized healthcare services; and any colleagues suggested by members of those organizations. Inclusion criteria for key informant interviews required that selected individuals had experience working on malaria activities.

#### Focus group discussions

Key informants identified specific urban and rural areas with the selected study sites for FGD locations and facilitated contact with local community leaders. The community leaders guided recruitment of community member participants for FGDs. Community members must not have participated in a FGD in another district in order to be included in the FGD.

#### Private provider interviews

**Fig. 2:**
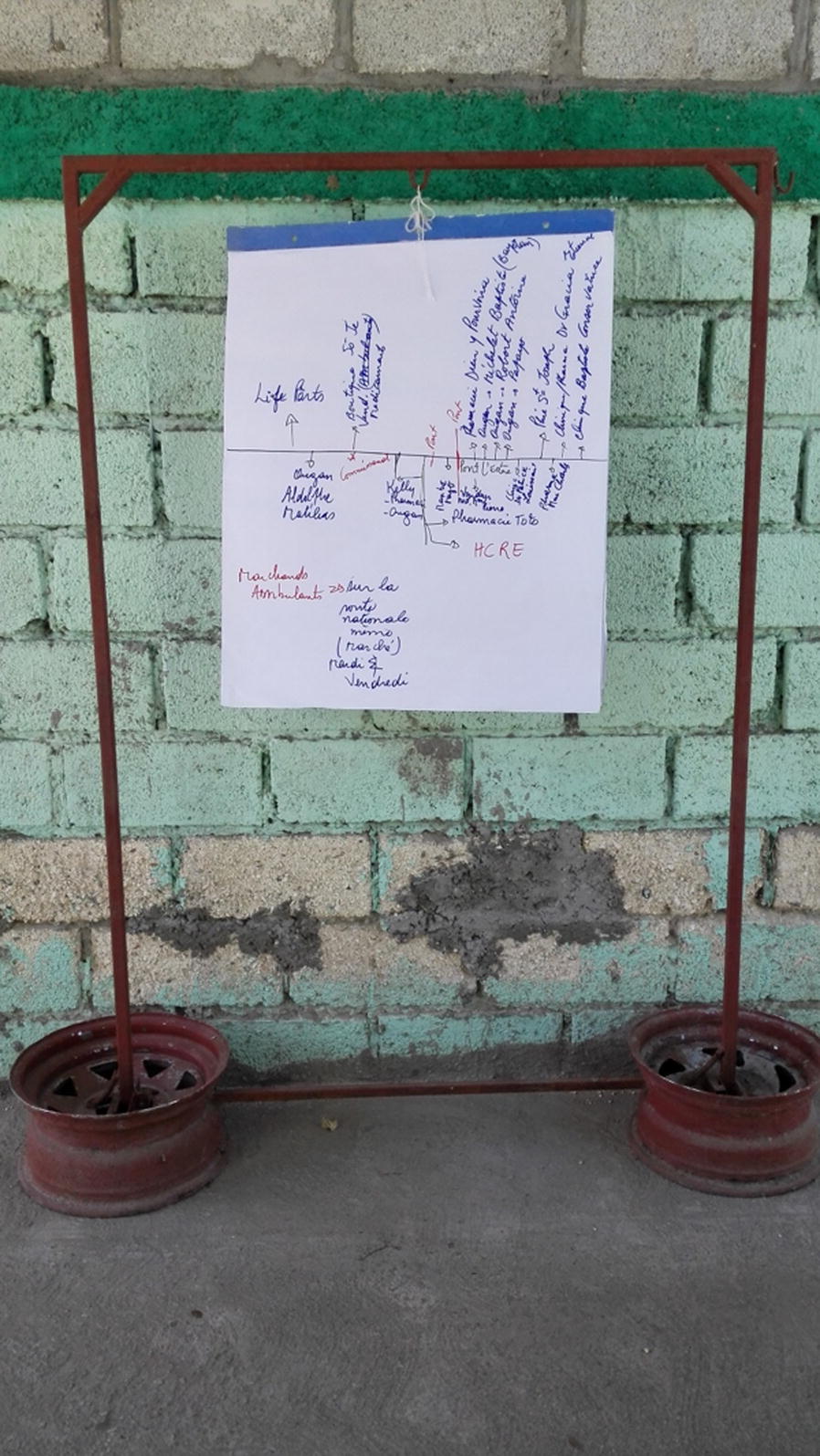
Example of private provider mapping exercise

FGDs obtained a local definition of the private health sector and mapped locations of local private providers to recruit for interviews (Fig. 2). Following the FGDs, community leaders guided recruitment of private providers. In order to be included, private providers must have fit the definition of “any outlet, facility, or person that provides clinical or diagnostic services and is not managed by a national or local government, including traditional healers” [6].

#### Patients of private provider interviews

The private providers interviewed assisted the study team by inviting patients seeking care at their facility to participate in an interview. All patients interviewed were currently seeking care for a fever from a provider interviewed on the day of the provider’s interview.

### Data collection

**Fig. 3:**
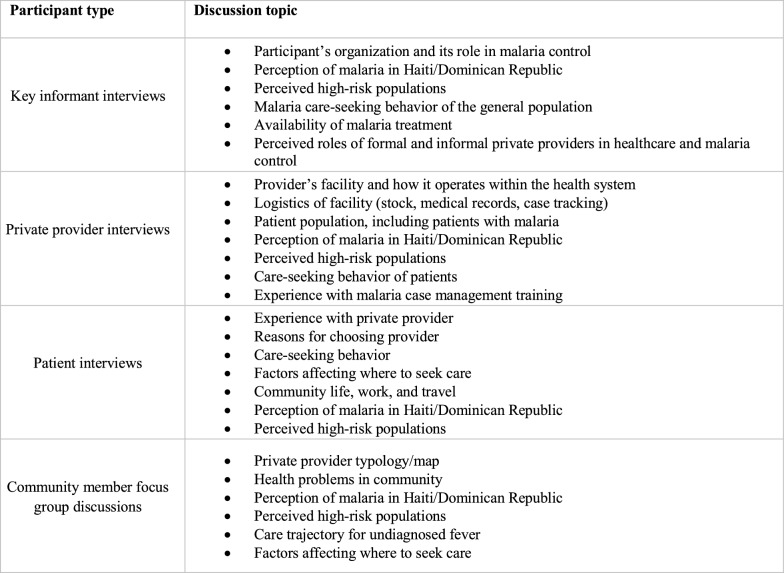
Discussion topics for interviews and FGDs

Data collection took place between December 2015 and February 2016 and was coordinated by staff at MOSCTHA in the DR, independent medical researchers in Haiti, and the University of California San Francisco (UCSF). Research assistants participated in a 3-day training in Port-au-Prince in Haiti and in Santo Domingo in the DR. The training covered the study protocol and qualitative methods in-depth. Following the main three-day training, research assistants participated in a 1-day refresher training conducted in each department. Research assistants conducted all research activities under the supervision of a local field manager and senior scientist in each country (JFL, JC). Interviews and FGDs followed semi-structured guides (topics in Fig. 3), lasted around 1 h, and were conducted in Spanish, French, or Creole depending on the location/participant. The interviews were audio-recorded, while the FGDs had a research assistant taking detailed notes.

### Data analysis

As the FGDs were used primarily to inform sampling of private providers, the notes were not formally analyzed. For the interviews, audio recordings were transcribed and then translated from Creole, French, and Spanish into English by staff at MOSCTHA, I-TECH, and an independent consultant. The transcriptions were uploaded into ATLAS.ti (Scientific Software Development GmbH, ATLAS.ti. version 8.1) for management and coding. Two researchers independently read a sampling of transcripts and developed preliminary codebooks for each participant group type (key informants, private providers, and patients). The researchers then compared codebooks, maintained open dialogue for all instances of discrepancy, and created a final codebook. The codebook was then applied to all transcripts, remaining flexible to allow for new codes as needed. Themes were generated by grouping codes within each participant type, noting both common threads and divergent opinions, and then were compared between participant types and countries. The themes were organized into areas of discovery, which, along with verbatim quotes, were used to produce the summary of findings.

### Ethical considerations

Ethical approval was obtained from the Institutional Review Boards of the Haitian Ministry of Public Health and Population, the Dominican Ministry of Public Health and Social Assistance, and University of California, San Francisco. Prior to all study activities, written informed consent was obtained from all participants in their native language.

## Results

The results are organized by country, with subheadings that correspond to four areas of discovery: (1) characterizing private sector providers and high-risk populations, (2) care-seeking behaviour of febrile individuals; (3) case management in private health sector facilities; and (4) barriers to and facilitators of private health sector engagement in malaria elimination efforts. Across both Haiti and the DR, there were 12 FGDs with 7–11 participants in each (98 participants total), 26 key informant interviews, 62 provider interviews, and 63 patient interviews (Tables 1, 2 and 3).

**Table 1:** Focus group discussions

Study site	Number of participants	Female	Male
Haiti	51	17 (33%)	34 (67%)
Artibonite Department	9	1	8
	11	0	11
Grande’Anse Department	7	4	3
	10	6	4
Nord/Nord-Est Departments	7	2	5
	7	4	3
Dominican Republic	48	32 (67%)	16 (33%)
Dajabon Province	8	6	2
	10	10	0
Monte Cristi Province	7	5	2
	8	5	3
Pedernales Province	8	3	5
	7	3	4
Total	99	49 (49%)	50 (51%)

All focus group discussion participants were 18 years of age or older

**Table 2:** Key informant interviews

Study site	Role	Organization
Haiti		
Artibonite Department	Departmental Coordinator	National Malaria Control Program
	Coordinator	Dessalines Unit Health Department
	Director	Bayonnais/Gonaives Health Center
	Medical Director	New Testament Health Center
Grande’Anse Department	Malaria Program Coordinator	National Malaria Control Program
	Staff	Universal Access Project
	Laboratory Technician	*Clinique La Paix*
	Laboratory Manager	Saint Antoine Hospital
Nord/Nord-Est Departments	Director	*Clinique Bethesda de Vaudreuil*
	Director	*Clinique ProFamille*
	Community Malaria Coordinator	*Centre Medico*-*Social de Ouanaminthe*
	Departmental Malaria Coordinator	Health Department, Northeast
Port-au-Prince	Head of Malaria Program	National Public Health Laboratory
	Head of Satellite Clinic Program	International Training and Education Center for Health
	Malaria Consultant	Ministry of Public Health and Population, Project Management Unit/Centers for Disease Control and Prevention
	Head of Monitoring and Evaluation	National Malaria Control Program
	Technical Advisor	Population Services International
Dominican Republic		
Dajabon Province	Director and Physician	*Hospital Matias Ramon Mella*
	Director	Radio Marien
	Provincial Manager	National Center for the Control of Tropical Diseases
	Director	Border Solidarity
Monte Cristi Province	Provincial Director	Ministry of Environment and Natural Resources
	Physician	Primary Care Unit
	Director and Founder	Mother Teresa Foundation
Pedernales Province	Head	National Center for the Control of Tropical Diseases
	Director General	Public Health Department

All key informants were 18 years of age or older

**Table 3:** Private provider and patient interviews

Study site	Female	Male	Total
Haiti			
Providers (All Haiti Sites)	19 (66%)	10 (34%)	29
Patients (All Haiti Sites)	19 (54%)	16 (46%)	35
Artibonite department			
Providers	6 (60%)	4 (40%)	10
For-profit clinic provider	1		
*Houngan*		3	
*Mambo*	1		
*Matrone*	1		
Nonprofit health centre provider	1		
Pharmacy staff	1	1	
Shop owner	1		
Patients	5 (42%)	7 (58%)	12
Grande’anse department			
Providers	6 (75%)	2 (25%)	8
For-profit clinic provider	1		
*Houngan*		1	
Itinerant drug vendor	1		
*Mambo*	3		
Pharmacy	1		
Patients	5 (50%)	5 (50%)	10
Nord/Nord-Est departments			
Providers	7 (64%)	4 (36%)	11
For-profit clinic provider	1		
*Houngan*		2	
Itinerant drug vendor	1		
*Mambo*	4		
*Matrone*	1		
Pharmacy staff		1	
Shop owner		1	
Patients	9 (69%)	4 (31%)	13
Dominican Republic			
Providers (All Dr Sites)	15 (45%)	18 (55%)	33
Patients (All Dr Sites)	15 (54%)	13 (46%)	28
Dajabon Province			
Providers	3 (27%)	8 (73%)	11
*Curandero* (traditional healer)		3	
For-profit clinic provider	1	1	
Pharmacy staff	1	2	
Shop owner	1	2	
Patients	5 (63%)	3 (37%)	8
Dominican	5	3	
Monte Cristi Province			
Providers	6 (60%)	4 (40%)	10
*Curandero* (traditional healer)	1	2	
For-profit clinic provider	2		
Pharmacy staff	1	1	
Shop owner	2	1	
Patients	4 (50%)	4 (50%)	8
Dominican	3	2	
Haitian	1	2	
Pedernales Province			
Providers	6 (50%)	6 (50%)	12
*Curandero* (traditional healer)	1	1	
For-profit clinic provider	1		
Natural medicine clinic provider	1		
Pharmacy staff		1	
Shop owner	3	4	
Patients	6 (50%)	6 (50%)	12
Dominican	3	4	
Haitian	3	2	
Total			
Providers	34 (55%)	28 (45%)	62
Patients	34 (54%)	29 (46%)	63

All private providers and patients were 18 years of age or older

## Haiti

### Characterizing private sector providers and high-risk populations

Six FGDs elicited the following categories of private health sector providers: private for-profit clinic providers, and private non-profit clinic and hospital providers, pharmacy owners, shop owners, itinerant drug vendors and traditional healers. Traditional healers include (but are not limited to): herbal doctors (‘*dokte feyes*’), Vodou priests (female ‘*mambos*’ and male ‘*houngans*’), senior Vodou priests (‘*badjikans*’), charlatans (spiritual healers that pose as doctors) and traditional birth attendants (‘*matrones*’).

Participants perceived migrants who travel for work to the DR or within Haiti to be at high risk for malaria. The majority of patients reported that there is a significant mobile population who work and live in different areas and that these migrants may contribute to malaria transmission. One patient from the Grande’Anse Department said that the people who are at highest risk of suffering from malaria are *“people who come from far away from this community, because they don’t protect themselves”* (Patient at non-profit private hospital, Grande’Anse Department). Other perceived high-risk populations included: slum populations, pregnant women, children, mountain-dwelling populations, agricultural/farm workers, fish farmers (in general and specifically noted in Grand Saline, a commune in the Artibonite Department), and populations living around rice paddies.

### Care-seeking behaviour

#### Self-medication

Self-medication, defined as taking medication without direction from a physician, with chloroquine, acetaminophen, paracetamol, or cotrimoxazole for an undiagnosed fever or suspected malaria was the most common first step in the care trajectory as reported by key informants, providers, and patients alike. One key informant said:

“*I think that there’s this tendency, as soon as the person has fever, they will go buy chloroquine…that’s what people do…there is an abuse of chloroquine because in general people take that for fever* (Key informant, Port-au-Prince).

Lack of accessibility to health sector facilities was reported as a barrier to seeking care and a facilitator of self-medication. One patient said:

*“If the person is not able to go to the hospital, his first reaction will be to seek a drugstore to buy medicines to help him feel better”* (Patient of traditional healer, Nord/Nord-Est Departments).

Medications, including chloroquine, were reported to be widely available in informal health sector facilities, including shops and itinerant drug vendors. Self-medication is very common across socioeconomic groups, but key informants believed migrant workers to be especially susceptible to self-medication due to the large presence of itinerant drug vendors positioned around bus stops and transit centres. When asked what type of person buys medication from itinerant drug vendors to self-medicate, a key informant explained:

*“People who travel [self*-*medicate], because they are getting in the bus to travel, and they [itinerant drug vendors] are persuasive. So voila, automatically people are going to buy that, [people] in bus stations who are going to travel out of the country to sell their products.”* (Key informant, Port-au-Prince)

#### Informal vs. formal health sector facilities

If symptoms persist after self-medicating, participants described a few different pathways of care-seeking behaviour. This decision depended on multiple factors, including quality of care and reputation of health facilities, socioeconomic factors, education, and personal or spiritual beliefs. Accessibility of health care facilities, however, was the primary factor. One key informant said:

*“It’s very easy [to get chloroquine] at the pharmacy. And the sellers even sometimes go buy chloroquine in a shop and sell it in the road. After [self*-*medicating], it depends on what care is offered in the zone. That can depend also on the education of the person. They can go look for traditional medicine, houngans. In the big cities, people prefer to go to private clinics.”* (Key informant, Port-au-Prince).

Individuals living in rural areas frequent traditional healers and public or private non-profit health centres, while those who live in larger cities may choose to seek care in formal private clinics due to their accessibility. If they had access to all types of facilities, patients said their decision would be based on perceived quality of care, cost, and spiritual beliefs. Middle- and high-income individuals, who may have insurance and can afford care at for-profit private clinics, may choose to pay the user fee as opposed to obtaining free treatment in public or mixed facilities due to the perceived higher quality of care and shorter wait times. Non-profit hospitals were generally preferred over public hospitals if given a choice, also due to perceived higher quality of care and shorter wait times at non-profit facilities. For example, one patient explains their choice to seek care at a non-profit hospital: *“*

*“The doctors acquire the ability to handle cases like malaria. They take care, they pay attention and they have medicines”* (Patient at non-profit hospital, Grande’Anse Department).

#### Traditional medicine

Traditional medicine was reported to be used by people of all socioeconomic statuses due to its accessibility as well as its cultural and spiritual relevance, but was reported to be especially common among the rural population. One patient explained they sought care from a traditional healer *“because I was in need to know my condition quickly”* (Patient of houngan, Grande’Anse Department). The long and costly journey to formal sector facilities is a barrier to seeking care in a hospital and encourages seeking care from local traditional healers. There were also many patient reports of receiving free or gifted medications or treatments from traditional healers, making traditional medicine a more affordable method of treatment. When asked to describe the care-seeking trajectory should symptoms continue, a patient at a traditional healer said:

*“If I’m in worse shape, I’ll see a doctor. The traditional healer is looking for a solution to get me a medical appointment, but I don’t have money. I haven’t seen a doctor since my sickness”* (Patient of traditional healer, Artibonite Department).

If fever symptoms progressed after an initial visit to a traditional healer, some patients reported they would return to a traditional healer for additional treatment while others reported they would choose to seek care in a formal health sector facility. This decision again weighed largely on access to formal health sector facilities and spiritual beliefs. For example, one febrile patient said:

*“I saw he [the traditional healer] provided an effective treatment for someone else…If I feel worse, I will go to a medical appointment or go to a church to pray”* (Patient of traditional healer, Grande’Anse Department).

Some patients reported “spiritual diseases”, where patients attributed fever and other symptoms to spiritual imbalances or witchcraft: *“This is not a common disease. It’s been thrown on me by humans”* (Patient of traditional healer, Artibonite Department). One patient reported seeking care first at a hospital, yet felt that the treatment approach wasn’t correct *“because I have a fetish disease”* (Patient of traditional healer, Grande’Anse Department).

Interestingly, when patients were asked where they would seek care if they *knew* they had malaria, the large majority of patients said they would seek care in a formal health sector facility. However, many of these same patients who reported they would seek care in a formal health sector facility if they knew they had malaria were visiting traditional healers for an undiagnosed fever at the time of the interview.

### Case management in private health sector facilities

#### Informal private providers

None of the informal private providers interviewed had RDTs or chloroquine-PQ bi-therapy. Some itinerant drug vendors and shop owners stocked chloroquine and reported selling it without a prescription. An itinerant drug vendor said:

*“I give him [a febrile individual] chloroquine or paracetamol then I recommend this person to go to a health centre”* (Itinerant drug vendor, Grande’Anse Department).

Traditional treatment methods for undiagnosed fevers included tea, roots, herbs, and bitter melon. Traditional healers explained that their knowledge is spiritually based. Nearly all traditional healers reported that they do not treat malaria and would refer a patient to a formal health facility if they suspected malaria. When asked if he would treat a case of suspected malaria, a traditional healer from the Nord/Nord-Est Departments said;

*“No. In that case I can seek your expertise. I’m in contact with medical doctors and nurses… I sometimes see illnesses here for which I must seek a doctor to add to my treatment”* (Houngan, Nord/Nord-Est Departments).

Patient interviews validated the traditional healers’ claim of referring patients to formal health facilities, with many patients reported being referred to formal sector hospitals by traditional healers in the past. Program-level key informants, however, generally did not seem aware of the willingness of traditional healers to interact with the formal health sector: *“Houngans do not refer patients to hospitals”* (Key informant, Port-au-Prince).

#### Formal private providers

Three of four formal private providers interviewed reported providing combination therapy to confirmed malaria cases. One private provider in the Grande’Anse Department did not have either chloroquine or combination therapy available. Only half of formal providers interviewed reported currently having RDTs in stock, which in turn made presumptive treatment relatively common in their practice. Accordingly, there were also a few patient reports of receiving presumptive treatment from formal private providers.

Key informants reported that non-profit facilities that have approval from the MSPP are provided RDTs and bi-therapy free of charge, in return for sending case and stock reports. One key informant explained this collaboration, saying:

*“The formal private sector, which has approval from the MSPP*, *has free access to the tests and drugs against malaria…they just have to send back their reports. The informal private sector is being challenged. They do not have access to the tests and drugs against malaria. This restriction included the ambulatory drug vendors and pharmacies. The latter ones have access to chloroquine, but not to primaquine”* (Key informant, Port-au-Prince).

One non-profit hospital provider reported collaborating with the MSPP, receiving training on malaria diagnosis and treatment as well as provision of RDTs and combination therapy. The other non-profit private hospital provider interviewed, however, reported no training from MSPP and no provision of RDTs and combination therapy. Key informants commented that the partnership between the MSPP and non-profit facilities is not as strong as it could be due to lack of finances, poor information dissemination from the government to the facilities, and logistical difficulties of returning case reports to the MSPP.

One non-profit private hospital and two for-profit private clinics reported that they commonly refer patients to receive malaria diagnosis with an RDT in a public hospital, with the instructions of returning with their test results to be treated.

*“We send them to a public hospital to do the lab test, then they come back with their prescription for the medicine”* (Non-profit private hospital provider, Grande’Anse Department).

However, other formal private facilities do not have access to anti-malarial medication and refer patients to the public sector for treatment.

*“When we are in front of cases like malaria, we refer them to [Public Hospital] to seek medicine, because we do not have a stock house here”* (For-profit private clinic provider, Grande’Anse Department).

### Barriers and facilitators to engaging the private health sector

Key informants unanimously agreed that traditional healers must be engaged in national malaria strategies because they are important community and spiritual leaders. The majority of traditional healers interviewed wanted to be integrated into the formal health system and were interested in participating in malaria elimination efforts. One mambo commented on the coexistence of the informal and formal medical systems

*“If we had a serious government we could do as some African countries do: traditional healers are part of the regular medical system, and are permitted to study cases at the hospital. I’ve been to many meetings that describe the existence of a combination health care system in African countries. Many cases at the hospital could be treated with herbs. Both systems are valuable. Your pills from the modern laboratories are effective but I can make some of the same remedies by pounding herbs. They can be just as effective and contain no chemically dead added elements. The remedy that has no chemical additives can have fewer negative effects and that is better for the patients”* (Mambo, Nord/Nord-Est Departments).

Traditional healers agreed that they would be motivated to report malaria cases in exchange for respect and acknowledgement of their practice within the formal health sector:

“[I would like] *that I be recognized as someone who helps them and who refers patients to them. They should value my work and be interested in my making a proper living”* (Matrone, Grande’Anse Department).

Ongoing trainings and incentives, such as pre-paid calling cards or money, were also reported to be motivating by traditional healers. Feelings about participating in national malaria elimination efforts among itinerant drug vendors diverged; some expressed interest, while others felt ostracized from the formal health sector and would hesitate to participate.

All formal providers were interested in reporting cases to the NMCP, with one non-profit health centre provider and one private clinic provider already reporting cases. Providers wanted additional staff, provision of RDTs and bi-therapy, and trainings to help with the responsibility of reporting cases. A private clinic provider explained:

*“We would have to afford employing and training the personnel necessary for such an endeavor, a Health Surveillance Officer to gather and analyze data for the ministry. Besides training, the element of human resource is fundamental, as are material resources all provided in a timely manner…I’m always interested to know how the health Ministry envisions malaria, what studies they have conducted to indicate the areas where it is most prevalent.”* (Private clinic provider, Nord/Nord-Est Departments)

### Dominican Republic

#### Characterizing private sector providers and high-risk populations

Six FGDs elicited the following categories of private health sector providers: private for-profit clinic providers, and private non-profit clinic and hospital providers, pharmacy owners, shop (*‘colmado’*) owners and traditional healers (*‘curanderos’*). Perceived high-risk populations included people who live in rural areas, people who work in the fields or near stagnant bodies of water, people who herd cattle, residents of Dajabon Province, and males in general due to their outdoor working conditions. Haitian migrants were also reported to be at high risk. One key informant said:

*“Most of our cases are from our neighbor, Haiti, because we live in a border region… The Healthcare system in Haiti is very weak, and when someone has malaria in Haiti, they think of witchcraft. And those who don’t believe in that [witchcraft] only use chloroquine for treatment.”* (Key informant, Pedernales Province).

#### Trajectory of care-seeking behaviour

The majority of patients interviewed reported self-medicating with acetaminophen at the first sign of a fever, prior to seeking care in a formal health facility. A patient at a pharmacy explained his decision-making process, saying:

*“Well, I decided to buy acetaminophen because you generally take it until you know if it is a virus or malaria* – *until you determine that it is malaria, you take acetaminophen*” (Patient at a pharmacy, Pedernales Province).

Many patients chose to purchase medication at *colmados* due to accessibility and the reliable availability of medications. A few patients believed that doctors would recommend acetaminophen anyway, so it is easier to buy it at a *colmado* instead of making a trip to the hospital.

*“You rarely go to the hospital because whenever you go to the hospital they just give you an acetaminophen when you have a fever, so, if I have fever and I know that the hospital will just give me an acetaminophen, I come to the pharmacy, buy it myself, and take it myself”* (Patient at pharmacy, Dajabon Province).

If symptoms persisted after self-medicating, most patients reported going to a formal health sector facility. Overall, programme-level key informants felt that public facilities were widely attended by the general population, with one key informant estimating that only 2% of people in Pedernales Province seek care at private facilities. Indeed, patients reported that they sought care at the local public sector primary care centre [Primary Care Units of the Ministry of Public Health (Spanish acronym UNAP)] if their symptoms do not resolve after self-medicating with acetaminophen. However, some patients sought care at private for-profit clinics after self-medicating. One patient explained her care trajectory, saying:

*“I went to a colmado and bought a pill, then I came to this doctor because I took the pills, but I felt the same. I went to the colmado when the fever began, 4* *days ago”* (Patient at a for-profit clinic, Pedernales Province).

The clientele of private clinics was reported by key informants to be middle- to high-income individuals, since private clinics typically require insurance. However, all five private clinic providers interviewed highlighted the large number of Haitian migrants who seek care in their clinics, and reported seeing patients coming from the public sector seeking a second opinion.

The perceived importance of traditional healers in the care-seeking trajectory of febrile individuals was low. One patient said:

“W*ell, I have never seen anyone go to a traditional healer…there are no traditional healers that people go to, and I think that [going] would be a mistake. In the hospital, I think, is the only place where there is medication to cure the disease”* (Patient at a for-profit clinic, Dajabon Province).

Key informants and patients agreed that that those who seek care from traditional healers do so mainly out of cultural or spiritual beliefs, and that it is relatively uncommon.

### Case management in private health sector facilities

#### Informal private providers

None of the informal private providers reported having RDTs or chloroquine-PQ bi-therapy, nor were they aware of the nationally recommended malaria treatment. While unaware of the recommended treatment, pharmacy owners were aware that treatment is available to patients within the public sector. *Colmado* owners reported selling analgesics (acetaminophen, Winasorb); nonsteroidal anti-inflammatory drugs (diclofenac, diclofex, ibuprofen, mefenamic acid, and aspirin); resfridol, a flu antiviral; ranitidine, an antacid and antihistamine; and omeprazole, a proton-pump inhibitor. The majority of traditional healers interviewed said they would refer a suspected case of malaria to a public health sector facility for testing, but first they would treat the patient with traditional medicine, such as herbal tea, homemade oral concoction, or a massage with oil.

#### Formal private providers

Formal private clinics ranged in perceived quality and services depending on their location. For example, private facilities in larger cities, such as Santo Domingo and Santiago, were perceived as high-quality facilities and as a prominent provider of health care delivery. However, the private sector plays a small role in malaria case management. A key informant explained how this service difference delays proper malaria case management:

*“Generally, in the cases of [malaria] mortality that we’ve had, people go to the private sector and after the private sector they go to the public and from there they determine that it is malaria”* (Key informant, Dajabon Province).

Private clinic providers reported seeing many patients with undiagnosed fevers, but perceived malaria cases to be very rare. None of the private clinic providers reported diagnosing a malaria case within the past year. However, four out of five private for-profit clinics had blood smear test supplies and reported sending samples to CENCET for malaria testing.

### Barriers and facilitators to engaging the private sector

Key informants emphasized the need for increased case reporting from private health sector facilities, and suggested the following means of engaging the private health sector: (1) sending a malaria technician from CENCET to visit private facilities to test patients with fevers for malaria and treat if positive; (2) campaigning/messaging to encourage private providers to help eliminate malaria; (3) workshops for private providers; and (4) promoting awareness of malaria elimination to the general population through television, radio, church networks, or neighborhood committees.

The majority of traditional healers and all pharmacy staff said they would be willing to report cases to the NMCP and would be motivated by participating in a workshop led by the NMCP. However, the lack of record keeping and lack of access to diagnostics were listed as significant barriers to case reporting.

## Discussion

This study employed qualitative methods to gain an in-depth understanding of malaria case management by private sector providers, care-seeking behaviour of at-risk individuals, and possible means of engaging the private health sector in malaria elimination efforts in Haiti and the DR. Key findings were: (1) self-medication was the most commonly reported first treatment-seeking behaviour for an undiagnosed fever in both Haiti and the DR; (2) seeking care from traditional healers for a fever is common in Haiti, either exclusively or in conjunction with care in the formal health sector; (3) most private providers, including traditional healers, reported an interest in participating in malaria elimination efforts, but emphasized the need for ongoing support and training. Programme-level key informants agreed with the need to involve private providers in malaria elimination efforts, especially traditional healers in Haiti; and (4) the Haitian migrant population was reported to be one of the most at-risk groups by participants from both countries.

The practice of self-medication has been found in many other similar settings [21–25]. Self-medication with chloroquine may contribute to chloroquine resistance if inappropriately dosed or an incomplete course is taken. Self-medicating malaria also affects case reporting. Communication on symptoms of malaria, proper care-seeking behaviour, and recommended treatment will be important for optimal surveillance and ensuring appropriate treatment is given.

This study also corroborates findings from a recent study in Haiti on the frequent use of traditional medicine for fevers, which can delay proper malaria case management in a formal health facility [26]. Nearly all patients in this study said that they would go to a formal health sector facility if they knew they had malaria, yet many were currently seeking care for an undiagnosed fever from a traditional healer and/or self-medicating with chloroquine. This suggests that individuals might not be linking the symptom of a fever to biomedically-defined malaria, and/or that there is stigma associated with traditional medicine. Prior studies in Haiti have shown that knowledge and awareness of malaria among community members is low, and that malaria is not considered as urgent or as important as other health issues [26, 27]. Given that education around etiology of disease has been shown to increase seeking care in formal sector health facilities in Haiti [28] and is formally recommended by the WHO [29], messaging around malaria symptoms, proper care-seeking behaviour, and treatment is recommended. Non-traditional methods of disseminating information may be necessary to reach the low-income population where television and radio ownership and newspaper readership are low [27].

Itinerant drug vendors and shops in Haiti commonly sell chloroquine without a prescription, despite the fact that a large majority of these vendors are not registered with the Haitian government nor are they owned or operated by a licensed prescriber or pharmacist [13]. Contrarily, virtually zero informal private providers in the DR have access to anti-malarial drugs, which can be largely attributed to the strictly enforced regulations. If regulatory policies are adopted and enforced alongside educational messaging, improved case management and surveillance is the anticipated outcome.

Engaging with traditional healers is an important part of private provider engagement in Haiti. These providers could provide referrals of febrile individuals to health facilities or community health workers, as has been observed in other settings [30–32]. The traditional healers in this study expressed interest in participating in national strategies, with some already reportedly referring presumed malaria cases to the formal health sector. Providing incentives such as cell phones, phone credit, or other subsidies could alleviate barriers to participation, and providing workshops or trainings could build trust between traditional healers and modern medicine providers and build the capacity of traditional healers to refer. However, providing trainings alone has been shown to produce only modest improvements of provider practices [33]. Providing ongoing support alongside trainings may lead to larger improvements, though further research with traditional healers and government stakeholders is needed in order to optimize the strategy, as has been done elsewhere [33, 34]. Involving community leaders in the recruitment and selection process of traditional healers to participate in engagement strategies has been recommended in a similar setting and could also be effective in Haiti [35].

There has been a countrywide effort to improve testing in Haiti with the rollout of RDTs in 2010. To continue this effort, the Haitian NMCP could continue distributing RDTs to formal private facilities, and possibly to select informal private facilities such as pharmacies or shops. A 2017 review found that expanding services of formal private providers to include malaria diagnostics has the potential to target anti-malarial drugs more effectively, but would require careful consideration of training and supervision of private providers [36]. Distributing RDTs to informal private healthcare providers, such as itinerant drug vendors, has also been shown to improve the uptake of RDTs and improve the number of correctly treated cases, especially together with price subsidies, education, and counseling [11]. Increasing distribution of RDTs and bi-therapy in Haiti, alongside adoption of a regulatory policy similar to the DR, could improve case management and reporting.

This study also emphasized the importance of addressing the Haitian migrant worker population. Legal and illegal immigration of Haitians and Dominican-Haitians into the DR has increased within the past decade due to political strife and natural disasters, and these migrants actively participate in the labor market and share tight spaces with lower-income Dominicans [37]. Thousands of people also cross the border daily for short term visits to buy and sell goods in Dajabon’s bi-national market [38]. In 2013, the Haitian and Dominican NMCPs collaborated on a community-based surveillance system pilot near the border that included active case detection followed by focal mass testing and treatment, and was successful at detecting and limiting an outbreak [18]. Continuing to collaborate around the border to better understand migrant population movements will be important in order to design a bi-national strategy that is targeted, effective, and able to achieve sustainable goals [2]. While specialized methods exist for surveillance of hard-to-reach populations like migrant workers (such as targeted border screening, peer-referral recruitment, and venue-based sampling) [39], further research is needed in order to determine the most effective and appropriate strategy for surveillance of the migrant population across the Haiti/DR border.

There are several limitations to this study. The distribution of providers selected in each study site was not necessarily representative of the actual distribution of types of providers in each site. Traditional healers in Haiti can be classified into many subcategories, such as *houngans* and *mambos*, but this study was unable to systematically distinguish them in this study and instead reported on “traditional healers” in general. Lastly, this study did not interview Haitian migrants living in the DR. Despite these limitations, this study is the first to provide an in-depth perspective of private health sector engagement in malaria elimination efforts in Haiti and the DR resulting in key takeaways to inform crucial next steps towards eliminating malaria on the island of Hispaniola by 2022.

## Conclusions

This research has important implications for engaging the private health sector into Hispaniola’s malaria elimination efforts. Key recommendations are: (1) direct clear, targeted messaging about malaria symptoms, care-seeking behaviour, and treatment to the general population and to migrant workers specifically; (2) increase distribution of RDTs and chloroquine-PQ to private health sector facilities, alongside the adoption of regulatory policies to improve malaria case management and reporting; (3) develop a specialized cross-border surveillance strategy targeting the migrant worker population; and (4) organize ongoing collaboration and trainings with private providers, including Haitian traditional healers, on case referrals and/or reporting.

## Data Availability

The dataset supporting the conclusions of this article can be requested from the authors.
